# Conditional Transgenesis Using Dimerizable Cre (DiCre)

**DOI:** 10.1371/journal.pone.0001355

**Published:** 2007-12-26

**Authors:** Nicolas Jullien, Isabelle Goddard, Samia Selmi-Ruby, Jean-Luc Fina, Harold Cremer, Jean-Paul Herman

**Affiliations:** 1 ICNE-UMR 6544 Centre National de la Recherche Scientifique (CNRS), Université de la Méditerranée, Marseille, France; 2 Centre de Transgenèse, Faculté de Médecine Nord, Institut Fédératif de Recherche (IFR) Jean-Roche, Marseille, France; 3 INSERM UMR 664, Faculté de Médecine RTH Laennec, Université Lyon I, Lyon, France; 4 IBDML-UMR 6216 Centre National de la Recherche Scientifique (CNRS), Université de la Méditerranée, Marseille, France; Katholieke Universiteit Leuven, Belgium

## Abstract

Cre recombinase is extensively used to engineer the genome of experimental animals. However, its usefulness is still limited by the lack of an efficient temporal control over its activity. We have recently developed a conceptually new approach to regulate Cre recombinase, that we have called Dimerizable Cre or DiCre. It is based on splitting Cre into two inactive moieties and fusing them to FKBP12 (FK506-binding protein) and FRB (binding domain of the FKBP12-rapamycin associated protein), respectively. These latter can be efficiently hetero-dimerized by rapamycin, leading to the reinstatement of Cre activity. We have been able to show, using *in vitro* approaches, that this ligand-induced dimerization is an efficient way to regulate Cre activity, and presents a low background activity together with a high efficiency of recombination following dimerization. To test the *in vivo* performance of this system, we have, in the present work, knocked-in DiCre into the Rosa26 locus of mice. To evaluate the performance of the DiCre system, mice have been mated with indicator mice (Z/EG or R26R) and Cre-induced recombination was examined following activation of DiCre by rapamycin during embryonic development or after birth of progenies. No recombination could be observed in the absence of treatment of the animals, indicating a lack of background activity of DiCre in the absence of rapamycin. Postnatal rapamycin treatment (one to five daily injection, 10 mg/kg i.p) induced recombination in a number of different tissues of progenies such as liver, heart, kidney, muscle, etc. On the other hand, recombination was at a very low level following *in utero* treatment of DiCre×R26R mice. In conclusion, DiCre has indeed the potentiality to be used to establish conditional Cre-deleter mice. An added advantage of this system is that, contrary to other modulatable Cre systems, it offers the possibility of obtaining regulated recombination in a combinatorial manner, i.e. induce recombination at any desired time-point specifically in cells characterized by the simultaneous expression of two different promoters.

## Introduction

The technology of gene inactivation through homologous recombination has, since its introduction in the late eighties, boosted immensely our understanding of the role of a great number of genes. However, it has been progressively realized that the interpretation of the results may be complicated by diverse factors such as differences among various inbred mouse lines, existence of compensatory adaptations, redundancies etc. Moreover, the modifications induced by the inactivation of a given gene at an early developmental stage (the most extreme modification being the lethality induced by the inactivation) may also interfere with, and make often impossible, the evaluation of a possible late role of that gene.

One way to avoid some of these problems is to have a temporal regulation of the inactivation and be able to induce it at any desired time-point. The introduction of the Cre/LoxP system has made this approach possible. Cre is a site specific recombinase that catalyzes the excision of a DNA segment flanked by two identical short (34 bp) asymmetric sequences, called LoxP, of same orientation, introduced into the genome by the experimenter. When that portion of DNA corresponds to a crucial part of the gene, the excision will lead to its inactivation [1]. Conditional inactivation has become a real possibility when solutions have been worked out for the *in vivo* regulation of Cre activity [2]–[5].

A first, “natural” approach to regulate Cre activity, and through it gene inactivation, is to put it under the control of a cell-type specific promoter. In that case one may obtain a spatial (cell-type dependent) regulation of Cre, that can even display a specific temporal pattern if the given promoter is activated only at a certain developmental stage or in a certain physiological situation [6]–[8].

The activity of Cre can also be dependent on an inducer given by the experimenter, and in that case one has the possibility of switching on Cre in any cell type. For that, two basic approaches have been worked out in the last few years: placing Cre under the control of a regulatable promoter or use a fusion protein to obtain a modulation of Cre activity by steroids.

The first option relies most of the times on the now classical tetracycline-regulatable system worked out by Bujard and his colleagues [9]–[12]. In that case the regulation of Cre is at the transcriptional level, and the reliability of the system is dependent on the tightness of the regulation of the promoter, i.e. its leakage when not activated, and the level of maximal activity after induction. Unfortunately, in many cases these systems are somewhat leaky, which means, for the utilization we are considering, that inactivation of the gene may occur spontaneously, before one would like to induce it experimentally.

The second approach is based on the fact that the fusion of the hormone-binding domain of the progesterone or estrogen steroid receptors with Cre will make the biological activity of the latter dependent on the steroid in the sense that the absence of the latter keeps the enzyme in a cellular compartment physically separated from the one in which its substrate is located [13]–[16]. Indeed, in the absence of the steroid, the fusion protein is sequestered in the cytoplasm of the cell by heat-shock proteins, and it is only following the addition of the steroid that the complex dissociates and Cre is able to enter the nucleus where it can combine with its substrate, i.e. chromosomal DNA, and exert its activity. Note that this approach can be combined with the previous one, allowing a combined cell-type dependent and temporal control of recombinase activity [17]–[19]. However, similar to the previous solution, these systems may be leaky. Moreover, induction of Cre activity within the central nervous system is not very efficient [13], [20], [21]. Last, but not least, the steroids that are used for induction can exert physiological effects of their own [22], [23].

We have recently developed a conceptually new approach to regulate Cre recombinase, that we have called Dimerizable Cre or DiCre [24]. DiCre is based on the functional complementation, using ligand-induced dimerization [25], of two inactive Cre moieties that can be associated by rapamycin through cross-linking the protein fragments FKBP12 (FK506-binding protein; [26]) and FRB, the ligand binding domain of FRAP (FKBP12-rapamycin associated protein; [27]) linked to the Cre moieties. DiCre has been validated *in vitro* and shown to be reliable, offering a low background activity and high-level induction resulting in characteristics that were better than that of a steroid-regulated form of Cre. Given its good *in vitro* performance, it represents a promising new tool that could offer an alternative to existing approaches for conditional transgenesis. Moreover, relative to other approaches, it offers the unique potential, being based on the coexpression of two separate Cre fragments, to achieve “combinatorial” or “intersectional” transgenesis [4]. The aim of the present work is to validate this tool for such *in vivo* uses.

## Results

### Targeting DiCre

DiCre requires the simultaneous expression in the cell of two inactive Cre moieties that can functionally complement each other. In our previous *in vitro* work, aimed at developing this system, we have examined several combinations of N- and C-terminal Cre moieties, differing in their length and/or in the linkers attaching FKBP12 (or FRB) to them [24]. This characterization indicated that these combinations differ in their background activity and/or the level of activity obtained following dimerization. We assumed that for conditional trangenesis it was important to aim at having the lowest possible background expression level, even at the price of having a somewhat lower induced activity. Thus, among the different combinations tested, the Cre59.F2/Cre60.F2 combination, that in our tests resulted in the lowest background, but still with a reasonably good activation level, was chosen for the *in vivo* tests of the system. As detailed previously [24], Cre59.F2 corresponds to the N-terminal moiety (aa 19–59) of Cre to which FKBP12 is fused through the flexible linker F2, while Cre60.F2 corresponds to the C-terminal moiety of Cre (aa 60–343) to which FRB is fused through the same flexible linker F2.

The expression of DiCre *in vivo* is complicated by the fact that this system requires the simultaneous expression of two constructs in the organism. With a classical transgenic approach that would imply a relatively tedious mating scheme using three mouse lines to bring the three constructs (the two components of DiCre plus the Cre-target construct) into the same animals. A way to simplify the experimental setup is to obtain a genetic linkage of the two components of DiCre. Rather than using a bicistronic construct and classical transgenesis, an approach that seemed less reliable, we decided, to achieve that aim, to express DiCre *in vivo* by homologous recombination, introducing a bicistronic construct containing its two components into a known locus. For that, we have chosen the Rosa-26 locus, that has been used extensively for several years to express foreign genes in mice, and for which extensive experience and molecular tools were available [28]–[30].

**Figure 1:**
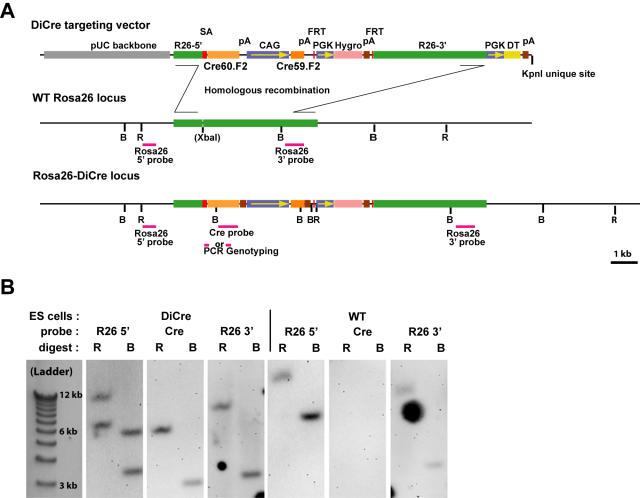
Targeting strategy for the creation of DiCre ES cells. A: Scheme of the targeting vector and of the Rosa26 locus following homologous recombination. “B” and “R” stand, respectively, for the BamHI and EcoRV sites that are used when screening ES cells clones for correct insertions using Southern blots and the probes indicated on the scheme. Fragments amplified when genotyping animals are also represented B: Representative examples of Southern blots obtained from wild type ES cells or clones with correct recombination, using the probes shown on the schemes above.

Details of the targeting construct are given on Figure 1A. Following its successful insertion, the C-terminal component of DiCre is expressed under the control of the Rosa-26 promoter, while the expression of the N-terminal component is controlled by the strong CAG promoter. In light of the ubiquitous expression and the absence of silencing of genes expressed from this locus, documented both during development and following birth [30], it was felt that this approach would ensure a generalized and reliable expression of DiCre. Note that a second promoter (PGK) with a downstream hygromycin-resistance gene, used for selection of successfully targeted ES cells, will also be present in the locus following targeting. However, this cassette has been removed secondarily from the genome by crossing F1 animals with Flpe-expressing mice [31] and is not present in the final lines presented here.

### DiCre activity in targeted ES cells

**Figure 2:**
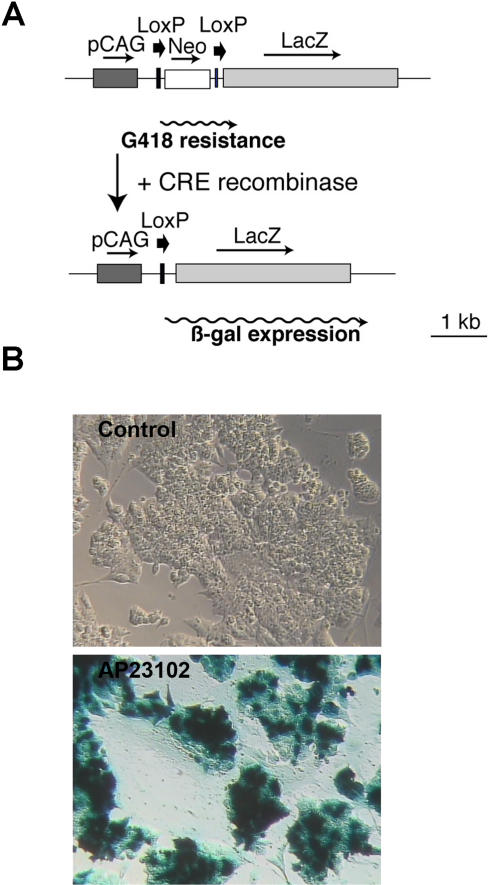
DiCre are functional in targeted ES cells. A: Scheme of the CALNLZ indicator construct used to test the functionality of Cre. B: ß-galactosidase expression, as revealed by the blue X-Gal reaction product, in ES cells with correct insertion of the DiCre construct into the Rosa26 locus and stable transfection of the pcDNA-CALNLZ construct. Cells are tested without (“Control”) or following exposure, for three days, to 20 nM of the dimerizer AP23102.

Following the obtention of clones of successfully targeted ES cells and before using them for blastocyst injection to obtain conditional deleter mice, we wished to test that DiCre was functional in these clones. Cells were transfected with an indicator construct, pcDNA3-CALNLZ [32] that expresses the ß-galactosidase reporter gene in transfected cells conditionally, dependent on the Cre-mediated excision of a neomycin resistance-STOP-pA cassette inserted between it and a CAG promoter (Fig. 2A). Following G418 selection, we obtained several subclones for the DiCre-containing ES clones. As shown on Figure 2B, there was a complete lack of expression of ß-galactosidase in the cells in the absence of inducer, indicating that background activity in these conditions is even lower than that we have observed earlier in fibroblasts. On the other hand, exposure of the cells to the inducer (20 nM of the rapamycin analog AP23102 for three days) led to the appearance of ß-galactosidase activity in close to 100% of undifferentiated cells, indicating that the system does work in ES cells. Note, however, that ß-galactosidase activity was lower or absent in flat cells, presumably engaged in some kind of differentiation pathway and surrounding the compact aggregates of undifferentiated ES cell. This aspect has not been pursued further.

Following this validation of the DiCre system, the initial targeted ES cells (i.e. not containing the indicator CALNLZ construct) were used for blastocyst injection. Several founder mice were obtained and used to establish mouse lines (called in the followings DiCre lines) after excision of the hygromycin-resistance cassette through mating of F1 mice with an Flpe-expressing deleter line.

### Characterization of DiCre in adult animals

**Figure 3:**
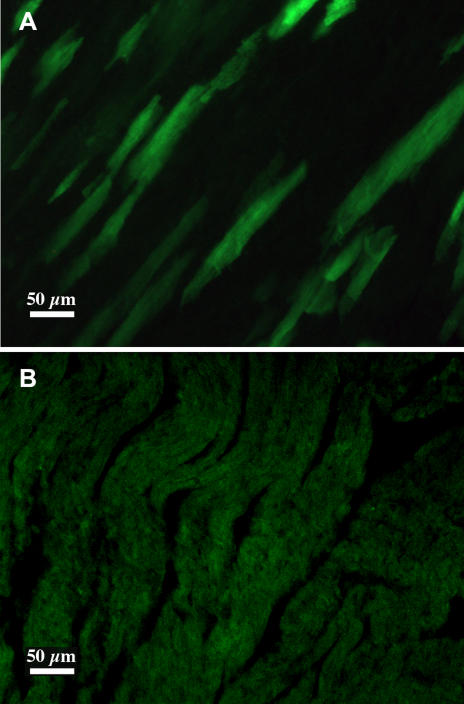
Induction of Cre-mediated recombination in DiCre×Z/EG mice. A: The presence of recombination is shown by EGFP expression in the heart of DiCre×Z/EG adult animals ten days after the end of treatment with the inducer (5×10 mg/kg rapamycin i.p.). B: In the absence of rapamycin treatment no specific fluorescence can be seen.

In a first series of test, DiCre mice were mated with Z/EG transgenic indicator mice to obtain double transgenic mice. The Z/EG transgene is similar to the CALNLZ construct depicted on Fig. 2A, except for the stuffer sequence that codes for LacZ and the gene expressed following the excision of the stuffer sequence that is EGFP [33]. Thus, cells of these animals are LacZ+ in the absence of recombination and green fluorescent following Cre-mediated excision. Young adult double transgenic progenies resulting from this mating were treated with the inducer (daily injection of 10 mg/kg rapamycin for five days) and sacrificed 5 days after the end of the treatment. In the absence of treatment no fluorescent cells could be detected on tissue sections prepared from a number of tissues (brain, pituitary, heart, lung, liver, kidney, skin, striated muscle). In treated animals EGFP-positive (as confirmed by immunohistochemistry, results not shown) green fluorescent fibers, indicating that recombination has taken place, were present only in the heart, and surprisingly, recombined green fluorescent cells could be detected in no other tissues (Fig. 3). Because of the low number of recombined cells, this switch on of EGFP expression could not be correlated to a significant decrease of the level of X-gal staining in the heart. Note that in untreated animals, strong and definite X-gal staining was observable only in the heart and muscle.

**Figure 4:**
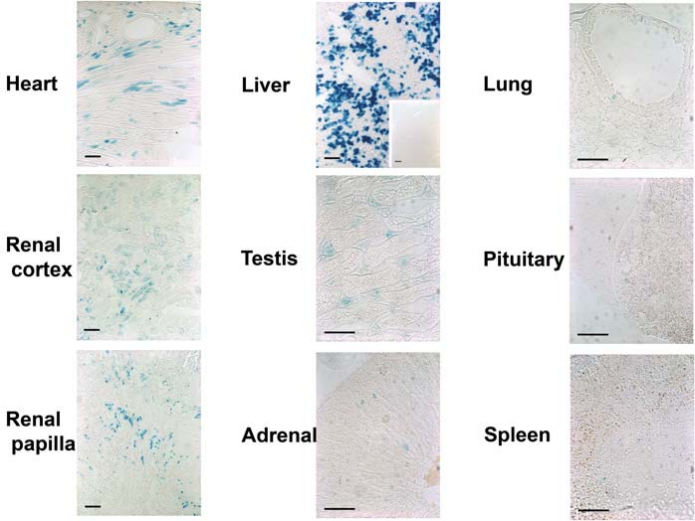
Induction of Cre-mediated recombination in various tissues of DiCre×R26R mice. The presence of recombination is shown by ß-galactosidase expression, as revealed by the blue X-Gal reaction product, in various tissues of adult DiCre×R26R animals ten days after the end of treatment with the inducer (5×10 mg/kg rapamycin i.p.). Bars represent 100 µm. The insert for the liver shows the total absence of recombination in the absence of rapamycin treatment.

To control and complement these results using another indicator mouse line, DiCre mice were mated with R26R animals [29] and the existence of recombination examined on tissue sections by X-gal staining. Again, no labeled cells were observed in the absence of treatment. On the other hand, and contrary to what had been observed with the Z/EG indicator line, recombination was widespread and could be observed in a number of tissues after treatment of the in DiCre×R26R animals with the inducer (Fig. 4). Thus, blue cells could be observed in heart, kidney, liver, testis, adrenals, fat tissue (not shown), lung, pituitary (adenohypohysis), spleen. However, the degree of staining differed greatly among these tissues, being quite dense in the liver where up to 30–40% of the cells were blue, intermediate in the heart and kidney, and low (only a few cells per sections) in the other tissues. Moreover, staining was not necessarily uniform in tissues with complex architecture, such as in the kidney where staining was more concentrated in the region of renal papilla and much less in the cortex, in the lung with staining detectable only in the alveolar epithelium, or in the testis, where only interstitial Leydig cells showed staining. No staining could be observed in the muscle, brain, or epidermis. The pattern of recombination was the same when higher doses of rapamycin were used (up to 40 mg/kg, results not shown).

**Figure 5:**
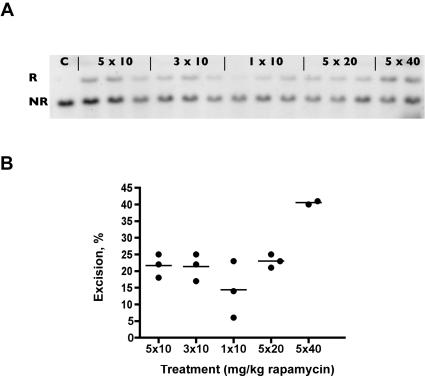
Dose-dependence of DiCre activation *in vivo*. A: Southern blots, using a LacZ probe, prepared from the liver of adult DiCreR26R animals ten days following 510mgkg, 310mgkg, 110mgkg, 520mgkg or 540mgkg i.p. rapamycin treatment, with two or three animals for each condition. The upper band R corresponds to the R26R allele after recombination, the lower band NR to the nonrecombined allele. B Quantification of the degree of recombination, as estimated by image analysis of the blot shown above.

To evaluate the number of rapamycin injections needed to induce DiCre-mediated recombination, mice were treated with different drug regimens, i.e. daily injections of 10 mg/kg rapamycin for one, three or five days. In parallel, higher doses (5×20 or 5×40 mg/kg) of rapamycin were also tested. Animals were sacrificed ten days after the last injection and recombination rate was quantitatively evaluated in the liver using Southern blots. The results indicate that the degree of recombination is equivalent for five or three injections and somewhat lower with a single injection (Fig. 5). It should be noted also that the use of the highest dose of rapamycin (40 mg/kg) seems to increase the degree of recombination.

### Characterization of DiCre during development

Although some recombination could be observed in DiCre×R26R progenies following rapamycin treatment during their intrauterine development (E13–14 or E18–19), its level was definitely lower (only a few cells per section) relative to that observed following postnatal treatment and restricted to the liver, kidney and heart (results not shown).

**Figure 6:**
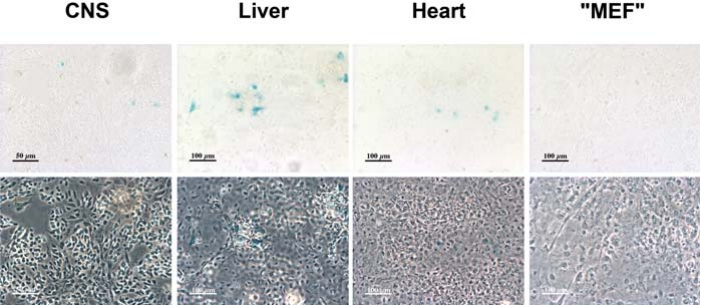
Induction of Cre-mediated recombination in primary cultures of embryonic tissues of DiCre×R26R mice. The presence of recombination is shown by ß-galactosidase expression, as revealed by the blue X-Gal reaction product on the bright-field (upper row) and phase contrast (lower row) images of the same fields, in primary cultures prepared from various tissues of DiCre×R26R E15 embryos. Cultures were exposed between 3 and 7 day *in vitro* to 10 nM AP23102.

To test whether the absence of recombination in embryos could be due to an insufficient passage of the dimerizer through the placental barrier, primary cultures were prepared from CNS, liver, heart of E15 DiCre×R26R embryos. Cultures were also prepared from the carcass of embryos using protocols described for obtention of Mouse Embryonic Fibroblasts [34] resulting in a mixed primary culture we call “MEF” and that contains, besides fibroblasts, other types of cells, such as myocytes and fused muscle fibers etc. Cre activity was induced by adding 10 nM AP23102 (DiCre×R26R cells) at 3 DIV and LacZ activity was tested four days later. Results were similar to what had been observed *in vivo*, i.e. some recombined cells could be observed among hepatocytes or cardiomyocytes obtained from DiCre×R26R embryos, but their proportion was very low (Fig. 6.).

### Expression of DiCre

**Figure 7:**
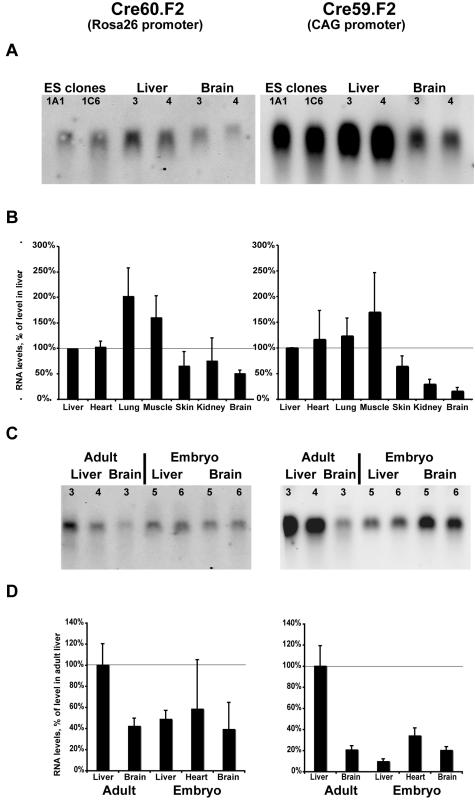
Expression of the DiCre constructs in tissues of adult or embryonic transgenic animals. A: Northern blots obtained from DiCre ES cells or tissues from young adult DiCre transgenic animals (two independent samples for each), indicating the level of mRNA for the indicated transgenes. B: Expression level, relative to that measured in the liver, of the same transgenes in tissues of adult animals, as measured by qPCR (means +/− SEM, n = 3–5). C: Northern blot, and D: qPCR analysis, as in panels 7A and 7B, from embryonic (E15) tissues and adult tissues used as comparison. Note that the adult tissues used here were not the same as those shown on panels 7A and 7B.

Evaluation of the level of transcripts, using Northern blots (Fig. 7A) or qPCR (Fig. 7B), in ES cells clones used to establish the DiCre mouse lines or in different tissues from young adult DiCre mice indicated that the level of transcription of Cre60.F2 from the Rosa26 promoter was relatively homogeneous and at comparable levels among these different tissues, except for the brain in which transcription seemed to be somewhat lower. For Cre59.F2 the level of expression, driven by the CAG promoter, was considerably higher than that mediated by the Rosa26 promoter for Cre60.F2. However, while for the Cre59.F2 transcript levels in the liver and ES cells were similar, the levels observed in the skin, kidney and brain were considerably lower. In tissues from E15 embryos, the level of Cre60.F2 transcription was diminished relative to the adult levels and transcription of Cre59.F2, controlled by the CAG promoter, was also found to be considerably decreased (Fig. 7C and 7D).

## Discussion

The aim of the present work was to assess the functionality of DiCre *in vivo* and its ability to be used to establish conditional deleter mice. Our results indicate that DiCre has indeed the potential to be used for conditional transgenesis and that DiCre mice could complement, and represent an alternative to, already existing approaches.

To be used for a general deleter strain, a modulatable Cre should fulfill several conditions. The two most important ones are, first, to have a low background activity, i.e. to induce no recombination in the absence of the inducer, and second, to lead, upon treatment with the inducer, to recombination in the different tissues of the animal. Additional conditions are that this induced recombination should be general within and among tissues, be independent of the developmental stage of the animals, and finally, that the inducer should not exert physiological effects on its own. It should be stressed that none of the existing systems to date fulfill all these criteria. Indeed, they often display some background activity, and their activation is usually mosaic, especially at later stages of development or in postnatal animals. Moreover, the most often used inducer, tamoxifen, has developmental and endocrine effects on its own that may present problems in some experimental situations [22], [23].

Results of the present study show that DiCre fulfills the first two conditions. Thus, we have not observed any recombination in the absence of treatment of the animals with rapamycin in the different tissues examined. This finding is in line with the fact that the DiCre variant that we have used to establish the DiCre mouse line corresponds to one that displayed a background activity among the lowest in our previous *in vitro* studies [24], and that it had no discernable background activity in ES cells (present study). Treatment with rapamycin, on the other hand, leads to recombination, albeit at different levels, in a number of tissues. On the basis of these findings, it can be concluded that DiCre represents a potential alternative for the creation of general deleter mouse strains.

It should be noted that a major advantage of DiCre over existing approaches is that, given its mechanism of action, its use may allow to create more complex experimental schemes. Indeed, using cellular promoters to control activity of Cre is a frequently used approach to restrict recombination to cells in which that promoter is active. In the case of DiCre, it will be possible to place each Cre moieties under the control of two different cellular promoters, targeting recombination to cells in which these two promoters are simultaneously active, and, moreover, superpose a temporal regulation to this targeting. The possibility of this kind of “combinatorial” or “intersectional” recombination, without the temporal regulation that DiCre allows however, was evocated in the case of the alpha-complementation of Cre fragments worked out a few years ago [35], but, contrary to DiCre, this system could not be shown to work *in vivo* (E. Casanova, personal communication). Note that a variant of this system, based on a leucine-zipper mediated unconditional complementation of Cre fragments has recently been shown to work *in vivo* [36]. However, comparison of this system with DiCre is not straightforward, as its performance has been documented only for the pancreas, where it led to a mosaic recombination. Moreover, given the unconditional character of the complementation, the recombination observed reflects recombination occuring during embryonic development, while DiCre could not be efficiently activated *in utero* (see also below).

While the present data demonstrate that DiCre works *in vivo*, they also show that induction of DiCre presents several aspects, related to the conditions listed above, that should be improved to ameliorate the system and make it more performant.

A first point is that the level of recombination brought about by the activation of DiCre was found to be highly variable both between and within tissues. This finding is not specific for DiCre, as it has been noted for all Cre constructs used to establish deleter mice. Thus, in similar experiments done with another deleter line expressing a tamoxifen-regulated Cre construct, we have found in adult animals a very similar pattern of recombination using the Cre-indicator lines used also in the present study (Jullien et al., submitted). This general variability might be related to the heterogeneity, between different tissues or in different cell populations of a given organ, in the characteristics of the indicator construct in its genomic context, such as a variable expression or silencing of the indicator transgene determined by neighboring host sequences, different local chromatin structures determining accessibility of the recombination target, etc. The same factors may also explain that different recombination patterns can be observed depending on the Cre indicator strain used. This influence of strains has been shown here by the major difference between our results obtained using the Z/EG and the R26R lines, but it has also been documented by other groups [20], [21], [37], [38].

An additional factor influencing the level of recombination (although not determining it strictly, as shown by the lack of clear correlation between the level of DiCre expression and the degree of recombination found in the different tissues, compare Figs. 4. and 7.) is the level of expression of the specific Cre construct used. This aspect may have a more important role in the case of DiCre, given that activation of DiCre is a ternary reaction involving rapamycin and the FKBP- and FRB-fused Cre moieties. Thus, it seems likely that to obtain an activity level comparable to that achieved using e.g. a classical steroid-activated version of Cre, a higher intracellular concentration of the DiCre components should be attained. This is especially true for the rate-limiting moiety of DiCre, which, in the present setup, corresponds to the Cre60.F2 fragment driven by the relatively weak Rosa26 promoter and that displays the lower level of transcription relative to the other moiety (see Fig 7.). This hypothesis might be explored in the future through the use of a promoter stronger than the Rosa26 promoter. A second parameter specific to the Cre constructs and that determines, in conjunction with the level of expression, the success of recombination, is its intrinsic activity. Thus, the different DiCre variants that we have tested previously *in vitro* have been observed to have different intrinsic activities [24]. As mentioned above, the variant tested here (Cre59.F2/Cre60.F2) possesses an intrinsic activity that is lower than that of other variants, but was chosen nevertheless for its low background activity and good activation ratio. Using a DiCre variant with a higher intrinsic activity (such as the Cre104.F5/Cre106.F5 combination, see [24]) might lead to a higher recombination efficacy *in vivo*, and that option will also be explored in the future. Note that using another DiCre variant presents the risk of having a somewhat higher background activity. In fact, there should be an interplay between the strength of the promoter and the DiCre variant used and it may well be that several combinations will have to be explored to find an optimal combination. Thus, the presently used variant, as controlled by the weak Rosa26 promoter displayed no background *in vivo*, while in our previous *in vitro* studies, when it was controlled by a strong viral promoter (CMV) it had some, even if quite low, background activity.

Finally, one might also invoke a possible variable distribution of rapamycin to explain the observed differences between tissues. However, insofar that the drug is known to be rapidly and evenly distributed among tissues [39], [40], this factor is unlikely to play a significant role in the observed variability.

DiCre-induced recombination during embryonic development was found to be very weak, if not absent, in most tissues. Our Northern blot analysis indicates that the level of expression of DiCre in embryonic tissues, especially its component controlled by the CAG promoter, is significantly lower than that found in young adults. The reasons of this are not clear, but it may explain the lack of recombination that we have observed both *in vivo* and in the *ex vivo* tissue culture conditions, and use of other promoters to control DiCre expression might solve this problem too. Note, however, that other factors might also contribute to the bad efficacy of DiCre in embryos. Thus, the bioavailability of rapamycin might be at an insufficient level in the embryos. Rapamycin is known to cross the placental barrier [41], but the level it reaches in the embryo is not known. However, our *ex vivo* culture experiment indicates that, while this factor may play a role, it may not be the most important and factors specific to the embryonic tissue might be involved. Thus, level of cellular uptake in embryonic cells might be not sufficient to ensure a high enough level of the drug within cells or, alternatively, the drug might be titrated out by the high endogenous FKBP12 level existing in embryos that could also, moreover, compete out the FKBP-linked Cre moiety ([42]; personal observations). The validity of these hypotheses will have to be evaluated in future experiments.

Besides these potential problems more specific to embryos, rapamycin presents other problems too. Indeed, this drug has, through its interaction with endogenous FRAP, numerous actions of its own: immunosuppression, interference with cell division, teratogenicity [43], [44]. Given these pharmacological actions, use of rapamycin analogs (rapalogs) devoid of such side effects would be highly desirable. Several such analogs, based on modifications of the FRB binding region of rapamycin, have been developed in the last few years [45]–[47]. They have in common not to recognize the endogenous target of rapamycin (FRAP), but to recognize a mutated form of this protein, such as the T2098L mutant used for the elaboration of DiCre, allowing its dimerization to FKBP12. While this mutation conserves the capacity of FRB to be dimerized also by rapamycin, it binds with high affinity these rapalogs, such as the AP23102 or AP21967 compounds that we have used for our *in vitro* characterization of the system. We have shown that these two drugs, while being good inducers of DiCre, have indeed less side effects insofar that they do not influence, contrary to rapamycin, cell division *in vitro*. This suggested to us that for *in vivo* uses they could represent a good alternative, devoid of adverse side effects, to rapamycin. Unfortunately, preliminary experiments have shown us that these two rapalogs are inefficient *in vivo*, as treatment of DiCre×R26R animals, be it during development or postnatally, induced, even at relatively high doses, considerably less recombination than rapamycin, due, maybe, to unfavorable pharmacokinetics of these drugs. Note that another rapalog, iRap, that is closely related to AP21967 [45], was found to activate DiCre in DiCre×R26R mice with the same efficacy as rapamycin (results not shown). However, insofar as it displays an affinity towards wild-type FRB that is comparable to that it has for the mutated form of FRB, it is likely to have the same side effects as rapamycin, and therefore cannot really be considered as a suitable option for the replacement of rapamycin.

Thus, alternative rapalogs will have to be tested to bypass the toxicity of rapamycin and improve these drug-related aspects of DiCre. A first option is to continue to explore drugs developed according to the logic presented above, i.e. drugs able to dimerize FKBP12 and a mutated form of FRB. A possible alternative is to modify the dimerizable partners fused to the Cre moieties themselves and use a mutated FKBP12 only, such as the F36V mutant, in conjunction with homodimerizer drugs that do not interfere with endogenous FKBP12 but have good pharmacokinetic characteristics [47]–[49]. Note that, due to the inability of these drugs to bind endogenous FKBP12, the latter option may present the added advantage of having a better efficacy during embryonic development (see above). It should be underlined, nevertheless, that while rapamycin seems to be quite toxic during development and in neonates, it seems to be well tolerated in older animals where, moreover, only one to three injections are required to activate DiCre, and thus it could be used, given appropriate controls, as an inducer of DiCre in adult animals.

In conclusion, DiCre represents a valuable new option to establish deleter mouse lines and achieve conditional transgenesis. It has interesting and unique characteristics, such as the possibility of achieving regulated combinatorial recombination. Future studies should help ameliorating it and make it a generally usable tool, in particular during development.

## Methods

### Plasmid construction

The targeting construct is depicted on Figures 1A. It was constructed by standard cloning techniques [50] and based on the pRosa26.1 targeting vector, donated by Dr. P. Soriano, for insertion into the Rosa26 locus. Cassettes inserted into this vector were the Cre60.F2 and Cre59.F2 sequences for the expression of DiCre [24], a hygromycin resistance cassette extracted from the p1710 vector (gift of Dr. J. Majors, St.Louis, MO), the PGK promoter from pRosa26.1, and the CAG promoter extracted from the pDRIVE-CAG vector (InVivogen, Toulouse, France). The adenoviral splice acceptor sequence was recovered from the pSA-ßgeo vector [51] donated by Dr. P. Soriano, the polyadenylation signals (SV40 pA downstream of the Cre59.F2 sequence and BGHpA downstream of the Cre60.F2 sequence) from pcDNA3.1 (Invitrogen, Cergy Pontoise, France).

The pcDNA-CALNLZ vector was constructed by inserting the CALNLZ cassette [32] into pcDNA3.1 in antiparallel orientation.

### ES cell culture and manipulation

Procedures for the manipulation of ES cells were based on published protocols [34]. R1 ES cells [52] were grown on mitomycin C-arrested STO fibroblasts expressing the neomycin and hygromycin resistance genes (gift of Dr. E. Robertson, San Diego, CA) in DMEM supplemented with 15% FCS and leukemia inhibitory factor (ESGRO, 1000 U/ml, Chemicon, Chandlers Ford, UK). Following electroporation of 10^7^ cells with 20 µg of the linearized targeting construct, selection was performed with 150 µg/ml hygromycin B for 8 days. Individual drug resistant clones were picked manually and transferred to 96-well microtiter plates, expanded and split. One of the resulting plates was frozen down and screening of clones was performed by Southern blots on the other plate, following digestion of isolated genomic DNA with BamHI or EcoRV and using “Rosa26-5′”, a 502 bp probe, located upstream of the insert and amplified from the Rosa26 locus using the primers 5′-GATAGGAACTGGAAAACCAGAGGA-3′ and 5′-GTAAGGGTCCAACAGAAAAGAGA-3′ (Fig. 1A.). A secondary Southern blot analysis, done on the positive clones of the first screen, allowed evaluating the integrity (using “Rosa26-3′”, a 709 bp probe located at the 3′ border of the insert) and unicity (using “Cre”, a 732 bp probe internal to Cre) of the insert. Location of the restriction site and probes, as well as a representative example of Southern blots, are indicated on Fig. 1. Correct clones were re-thawn, expanded and frozen down till their further use. Chimeras were produced by injection of ES cells into blastocysts obtained from C57Bl/6 mice (Charles Rivers, Lyon, France)

To test functionality of the regulatable Cre constructs inserted into the Rosa-26 locus, recombined ES cell clones were re-transformed by electroporation with the linearized pcDNA-CALNLZ construct, selected with 250 µg/ml G418 for one week and subcloned before testing.

### Primary culture

Primary cultures were prepared form E15 embryos. Dissected liver and CNS tissues were digested 15 mins at 37°C in trypsin-EDTA, heart 30 mins at 37°C in trypsin-EDTA followed by 30 mins in collagenase. Following digestion, tissues were mechanically dissociated in culture medium containing 0.01% DNAse and the cells were plated onto polylysine-coated coverslips in DMEM/F12 (liver and neural cultures) or DMEM (heart cells) containing 10% FCS. Mixed “MEF” cultures were prepared from the carcass of the embryos digested for 45 mins in trypsin-EDTA with intermittent shaking, dissociated and plated onto polylysine-coated coverslips in DMEM with 10% FCS. Cells were grown in 5% CO_2_:95% air at 37°C for three days before addition of the inducers, grown for further 4 days and fixed using 2% paraformaldehyde in phosphate buffer (0.1M, pH 7.4). ß-Galactosidase activity was revealed by incubating the fixed cells in PBS containing 1 mg/ml X-Gal, 5 mM potassium ferricyanide, 5 mM potassium ferrocyanide, and 2 mM MgCI_2_.

### Animal handling

Animals were maintained in standard conditions, with food and water *ad libitum* and a 12 h∶12 h dark∶light photoperiod. The hygromycin cassette from F1 recombinant DiCre mice was eliminated by crossing with FLPe mice [31]. Mice were then backcrossed for 2 generations onto C57Bl/6 background and then, to facilitate subsequent characterization, intercrossed to obtain homozygote lines. Mice were genotyped for the presence of the transgene by PCR on tail DNA obtained by the “hot-shot” method [53].

Homozygote recombinant mice were mated with heterozygote Z/EG [33] or homozygote R26R [29] Cre indicator mice. Progenies born from mating with Z/EG mice were genotyped for the presence of the Z/EG transgene by PCR on tail DNA.

Rapamycin (Sigma, St. Louis, MO, or LC Labs, Woburn, MA) or the rapamycin analogs AP-23102 or AP-21967 (Ariad Inc., Boston, MS) were dissolved in N,N-dimethylacetamide (DMA) and then diluted in a mixture of 4% DMA, 10% polythylene glycol (average MW 400) and 17% polyoxyethylen sorbitane monooleate (Tween 20). Injections were given intraperitoneally (i.p.).

### Histology

Animals were deeply anaesthetized and sacrificed by transcardiac perfusion of saline followed by 2% paraformaldehyde in phosphate buffer (0.1 M, pH 7.4). When X-gal staining was to be performed, the fixative contained 1 mM EDTA and 2 mM MgCl_2_. Following 3–5 h postfixation at room temperature, tissues were cryoprotected overnight at 4°C in 30% sucrose and frozen down for preparation of 25 µm cryostate sections. EGFP expression was examined by direct inspection of sections or following immunostaining using a polyclonal anti-GFP antibody (Molecular Probes, Invitrogen, 1/5000) followed by a secondary, Alexa Fluor 594-labelled, anti-rabbit antibody (Molecular Probes, Invitrogen, 1/600). ß-Galactosidase expression was revealed by incubation of the section in PBS containing 1 mg/ml X-Gal, 5 mM potassium ferricyanide, 5 mM potassium ferrocyanide, 2 mM MgCI_2_, 0.01% sodium deoxycholate, and 0.02% Nonidet P-40.

### Northern blots and qPCR

Five µg total RNA, extracted by Trizol (Invitrogen) for tissues or RNEasy (Qiagen) for cultured cells, were loaded onto a formaldehyde/MOPS agarose gel, and transferred after migration onto Hybond N+ membrane (Amersham). The Rosa26 driven Cre60.F2 transcript was revealed by hybridization with a 732 bp digoxigenin-labeled probe specific for C terminal Cre moiety; the CAG promoter-driven Cre59.F2 transcript was hybridized with a 363 bp digoxigenin-labelled fragment of the Cre59.F2 CDS. Hybridization, washing and revelation using CDP* were adapted from Roche's DIG membrane protocols.

Reverse transcription of 1 µg RNA was done using SuperScript II and random primers as indicated by the manufacturer (Invitrogen). qPCR was done using the SYBR-Green PCR master mix (Applied Biosystems) and run onto an ABI Prism 7700 station. Specific primers were designed with AmplifX (http://ifrjr.nord.univ-mrs.fr/AmplifX). Calculations were done by the ΔΔCt method [54] using 18S RNA as reference, after having determined, using dilution series, that amplification efficiencies were homogeneous for the different primer pairs and very close to 100%.

### Southern blots

To quantify the degree of recombination of the R26R reporter allele, genomic DNA was prepared from tissues following proteinase K digestion, using standard procedures [50]. Following digestion by HindIII, that was determined previously to allow discrimination of recombined and non-recombined R26R alleles, migration and transfer to Hybond N+ membranes, Southern blots were completed using a digoxigenin-labelled 890 bp for LacZ. Blots were digitalized using a ChemiGenius II station (Syngene, Cambridge, UK) and quantification of the native 16 bits gray level files was performed using GeneTools (Syngene).
